# Therapeutic Effects of *Nigella sativa* Oil and Whole Seeds on STZ-Induced Diabetic Rats: A Biochemical and Immunohistochemical Study

**DOI:** 10.1155/2024/5594090

**Published:** 2024-08-10

**Authors:** Naif AlSuhaymi

**Affiliations:** Department of Emergency Medical Services Faculty of Health Sciences AlQunfudah Umm AlQura University, Makkah 21912, Saudi Arabia

## Abstract

**Background:**

Type II diabetes mellitus (DM) is an increasing health problem that has negative impacts on patients and healthcare systems, worldwide. The development of new therapies with better efficacy, fewer side effects, and lower prices are urgently needed to treat this disease.

**Aim:**

To evaluate and compare the therapeutic effects of *Nigella sativa* (*N. sativa*) seed and oil on the biochemical parameters and regeneration of pancreatic islets (or islets of Langerhans) of streptozotocin (STZ)-induced diabetic rats.

**Materials and Methods:**

The diabetic rat model was prepared by administering a single dose of STZ (35 mg/kg body weight). The whole seed or the oil of *N. sativa* was administered to the diabetic and control groups for a period of 28 days, but not to the negative and STZ controls. Serum blood glucose, liver enzymes, lipid profile, and renal function tests (uric acid, albumin, total protein, urea, and creatinine) were measured in all groups. After the rats were euthanized, their pancreases were extracted, and then sectioned and fixed on slides in preparation before staining with H&E stain and immunohistochemical study.

**Results:**

Treatment of STZ-diabetic rats with *N. sativa* seeds or oil significantly improved their serum glucose levels, lipid profiles, and liver and renal functions as well as preserved the integrity of pancreatic *β* cells.

**Conclusion:**

*N. sativa* seeds and oil demonstrate significant therapeutic improvement effects on DM and its related complications including effective protection of islets of Langerhans. The therapeutic benefits of *N. sativa* seeds and oil on DM and its related complications are comparable.

## 1. Introduction

Diabetes mellitus (DM) is considered one of the top five causes of death, worldwide [1]. The prevalence of DM is increasing worldwide; in the Kingdom of Saudi Arabia (KSA), the number of cases is steadily increasing, and its complications are a major health problem [2, 3, 4]. Several studies in human and animal models with DM have shown changes in the antioxidant status of cells [5, 6, 7, 8, 9], as well as disturbing influences on the defense system [10, 11, 12]. Evidence has also been found that increased glucose concentration can suppress natural antioxidant defense such as vitamin C and glutathione [13]. In addition, such oxidative stress can lead to the development of type I diabetes mellitus by inducing apoptosis *β* Langerhans's cells and increasing insulin resistance [14, 15].

Black cumin (*Nigella sativa* or *N. sativa*) is an annual flowering plant of the Ranunculaceae family native to Southwest Asia, that is commonly used in many foods and medicines [16, 17, 18, 19]. *N. sativa* seeds and oil are frequently used for the treatment of many diseases, including rheumatoid arthritis, asthma, inflammatory diseases, and DM [19, 20, 21, 22, 23]. The World Health Organization (WHO) estimates that approximately 80% of people benefit from herbal remedies; therefore, it is necessary to evaluate the rich heritage of traditional medicine [3]. In KSA, specifically, medicinal plants have been widely used in traditional medicine [24, 25]. However, only a few plant species have been thoroughly studied for their therapeutic properties, mechanism of action, safety, and toxicity, meaning that much research on medicinal plants remains to conducted.

South Asia is a rich source of herbs and plants with medicinal value, and *N. sativa* is one such herbal product; it has been used as a spice since ancient times, with a well-known medicinal value for treating various diseases, due to its antibacterial [26] and antidiabetic properties [27]. Moreover, the safety of *N. sativa* extracts has been extensively studied and proven to have an extensive margin of safety for therapeutic doses, with no harmful effects on the heart, liver, kidneys, and pancreas when used in food supplements and dietary adjuvants [28, 29, 30, 31].

People with glucose intolerance can obtain great therapeutic benefits from *N. sativa* since it augments insulin secretion after glucose consumption with reduction in the amount of glucose absorption from the intestinal mucosa [32, 33, 34]. Moreover, *N. sativa* could improve the damage that may affect *β* cells of the pancreas after being exposed to toxic elements, i.e., cadmium [35].

Streptozotocin (STZ) is a naturally occurring nitrosourea [36] that is frequently used for induction of insulin-dependent DM because of its toxic effect on Langerhan's *β* cells resulting in its irreversible damage with failure in insulin secretion [36, 37].

The present study investigated the effect of the whole seed and oil of *N. sativa* on the islets of Langerhans of diabetic rats treated with STZ, as well as on their serum glucose level, lipid profiles, and liver and renal functions.

## 2. Materials and Methods

### 2.1. Experimental Design

Thirty-six male Sprague–Dawley rats, aged 10–12 weeks and weighing 200–250 g, were bought from the Animal House at the National Research Centre in Cairo, Egypt, and were divided into six groups of six animals each as follows: group I, negative control—normal rats fed on a standard synthetic diet; group II—normal rats fed on standard synthetic diet supplemented with *N. sativa* seed powder (150 mg/kg body weight) daily for 4 weeks by intragastric intubation; group III—normal rats fed on standard synthetic diet supplemented with *N. sativa* oil (5 ml/kg body weight) daily for 4 weeks by intragastric intubation; group IV—STZ-diabetic rats fed on standard synthetic diet; group V—STZ-diabetic rats fed on standard synthetic diet supplemented with *N. sativa* seed powder (150 mg/kg body weight) daily for 4 weeks by intragastric intubation; group VI—STZ-diabetic rats fed on the standard synthetic diet supplemented with *N. sativa* oil (5 ml/kg body weight) daily for 4 weeks by intragastric intubation. All animals were housed in a standard laboratory with individual cages with a solid floor in a chemical-free room with a controlled room temperature (23 ± 2°C), 40%–60% relative humidity, and artificial lighting (12 hr dark/light cycle), all animals were fed with designated meals and offered water to acclimatize and ensure average growth and behavior.

### 2.2. Induction of Diabetes in Rats

A single administration of streptozotocin (STZ) (Sigma, St Louis, MO, USA) (35 mg/kg body weight) dissolved in a 50 mM cold citrate buffer (pH 4.5) was used to induce diabetes [38]. STZ was administered intraperitoneally on the first day of the experiment; blood glucose levels were determined after 2 days to ensure that the rats developed DM.

### 2.3. Diet Composition

Casein (150 g/1 kg diet), unsaturated fats (100 g/1 kg diet), sucrose (220 g/1 kg diet), maize starch (440 g/1 kg diet), cellulose (40 g/1 kg diet), a salt (40 g/1 kg diet), and a vitamin mixture (10 g/1 kg diet) ultimately formed the basic synthetic diet. The mixture of salt and vitamin mixture was developed using the AIN-93M diet as a guide [39]

The black cumin seeds of *N. sativa* were purchased from a local market in Cairo. The seeds were ground into powder with grinder and dissolved in freshly prepared carboxymethyl cellulose and each animal belonging to the two seed groups received this mixture daily at a dose of 150 mg/kg body weight by intragastric intubation. *N. sativa* oil was prepared under sterile conditions by cold pressing method of the Prime Natural Organic Black Seed.

### 2.4. Collection of Blood Samples

During the 4-week experiment, the animals were made to fast for 12 hr before collection of bold samples for blood glucose level and other biochemical analysis. Collection of blood samples was performed under sodium pentobarbital anesthesia (50 mg/kg, i.p.) from the retro-orbital venous plexus at the first, seventh, and fourteenth days as well as the end of experiment. At 28 days, one blood sample was collected from each animal at a time, after which serum and plasma were separated by centrifugation (Sigma Laborzentrifuge GMBH, Germany, model 2-153360 osterode/Hz) at 4,000 rpm for 15 min and stored at −20°C.

### 2.5. Biochemical Parameters

Biochemical evaluation of the serum glucose [40]; lipid profile including total cholesterol, triglycerides (TG), low-density lipoprotein (LDL), and high-density lipoprotein (HDL) [41, 42, 43]; liver function including aspartate aminotransferase (AST), alanine aminotransferase (ALT), and alkaline phosphatase (ALP) [44]; and renal function including creatinine, urea, and uric acid [45] was performed using standardized enzymatic procedures, spectrophotometrically (Roche Cobas 400, Mannheim, Germany).

### 2.6. Histology

Autopsy specimens were collected from the rats' pancreases of the different groups and fixed in 10% formalin for 24 hr. The samples were dehydrated with ascending dilutions of ethyl alcohol. Then cleaned in xylene and embedded in paraffin wax for 24 hr at 56°C in a hot air oven. The paraffin wax tissue blocks were cut into 4 *µ*m-thick sections using a sled microtome. The obtained tissue sections were removed by rotary microtome, deparaffinized, and stained with hematoxylin & eosin (H&E) [46] for examination with a light microscope (Leica).

### 2.7. Immunohistochemistry Testing

Pancreatic paraffin sections were dewaxed in xylene and then dehydrated for immunohistology examination. Then, 4 *µ*m-thick sections were hydrated through PBS and subjected to primary guinea pig anti-insulin antibodies (Abcam US, 1 : 1,000), then incubated overnight at room temperature; thereafter, the sections were washed twice in TBS plus 0.025% Triton X-100 (Abcam US) for 5 min and blocked with 10% normal horse serum (Abcam US) with 1% BSA in TBS for 2 hr at room temperature. Subsequently, the sections were incubated with the secondary antibody sections of the goat antirabbit horseradish peroxidase (HRP) antibody (Abcam US, 1 : 500). The reaction was detected with DAB/H_2_O_2_ for 2–5 min and counterstained with hematoxylin.

### 2.8. Morphometric Study

Histological image analysis of pancreatic sections was performed using the ImageJ software which can analyze biological systems on various scales, from structural details determination of number of cells, area, localization, and concentration [47].

### 2.9. Statistical Analysis

All the obtained data from the biochemical analysis, and the histopathological study were analyzed using the Statistical Package for the Social Sciences (SPSS) software version 26 (IBM, Armonk, NY, USA). Unpaired *t*-test was used for comparison of quantitative data before and after treatment with *N. sativa* while the paired *t*-test was used to compare the effects of *N. sativa* seeds or oil-treated groups compared to the normal control or STZ diabetic groups. ANOVA was used when comparing the effect of *N. sativa* seed or oil on the blood biochemical parameters of the six examined groups. Probability values < 0.05 were considered statistically significant.

## 3. Results

### 3.1. Blood Glucose Levels and Biochemical Parameters

Our findings confirmed that using *N. sativa* seed and oil was safe. Compared with the negative control group I, all the parameters remained within normal figures with no significant changes in the normal seed and oil-fed groups II and III. However, the STZ seed-treated (V) rats and the STZ oil-treated (VI) rats showed significant differences in their glucose levels, renal function, liver functions, and lipid profile tests compared to the STZ (IV) nontreated rats.

**Figure 1:**
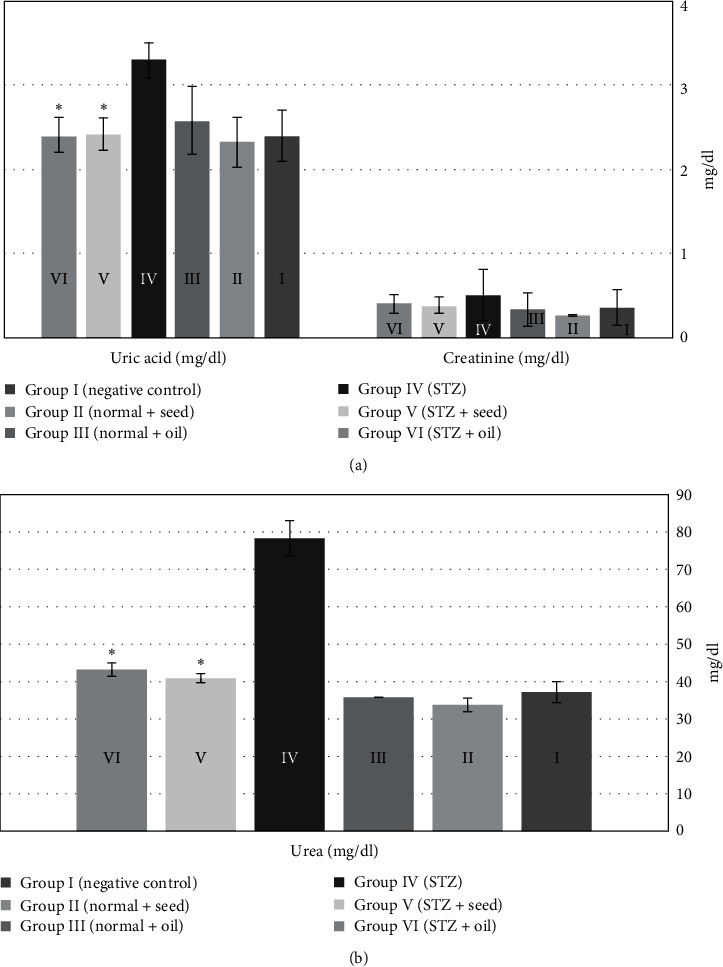
The serum levels of uric acid, creatinine (a), and urea (b) in rats in the six studied groups after 28 days of the experiment.  ^*∗*^*p* < 0.001.

**Figure 2:**
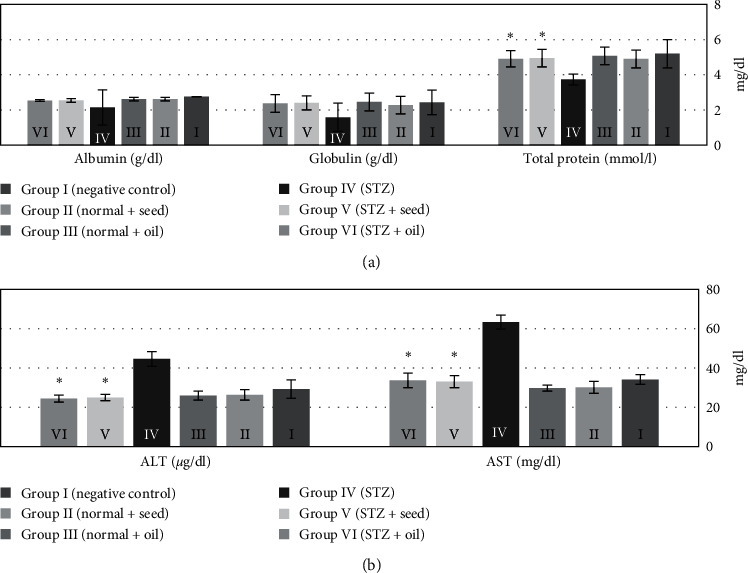
The serum levels of albumin, globulin, total protein, alanine transaminase (ALT) and aspartate transaminase (AST) levels in rats in the six studied groups after 28 days of the experiment: (a) Liver function (proteins) and (b) liver function (liver enzymes).  ^*∗*^*p* < 0.001.

**Figure 3:**
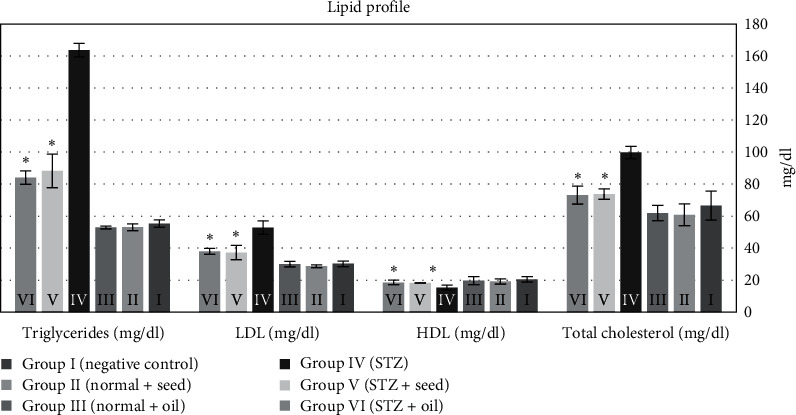
The serum levels of triglycerides, LDL, HDL, and total cholesterol in rats in the six studied groups after 28 days of the experiment.  ^*∗*^*p* < 0.001 for triglycerides,  ^*∗*^*p* < 0.01 for total cholesterol,  ^*∗*^*p* < 0.001 for LDL, and  ^*∗*^*p* < 0.05 for HDL.

Comparison between the negative control group and the STZ group on all biochemical parameters was performed to confirm the appropriateness of the diabetes induction. All the parameters showed significant differences indicating the negative impacts of DM in the STZ group compared to the normal figures as measured in the negative control group (Figures 1, 2, and 3).

The results in Table 1 show that after induction of diabetes with STZ, there was a significant increase in blood glucose level at day 7 in the diabetic groups, i.e., group IV (414.1 ± 1.6), group V (408.3 ± 1.7), and group VI (412.2 ± 1.4) over that of the control group I (99.4 ± 0.3), and the nondiabetic seed and oil treated groups II and III, (*p* < 0.01). However, neither significant difference was found among control groups I, II, and III nor among diabetic groups IV, V, and VI.

On day 14, there was significant decrease in blood glucose levels in group V (234.5 ± 0.6) and group VI (233.3 ± 1.1) compared to group IV (414.5 ± 1.7) (*p* < 0.01). However, compared to the initial blood glucose levels, the day 14 blood glucose levels of groups I, II, and III showed no significant change.

On day 28, there were significant decreases in blood glucose levels in group V (156.3 ± 0.8) and group VI (157.1 ± 0.4) compared to group IV (414.8 ± 1.3) (*p* < 0.001).

Notably, there was no significant difference between the blood glucose levels of groups V and VI on days 7, 14, and 28.

The serum levels of uric acid and urea were significantly increased after induction of DM in group IV compared to group I (using paired *t*-test; *p*  < 0.001); however, these levels were significantly reduced after treatment of STZ rats with *N. sativa*, either with seeds (group V) or oil (group VI), (using paired *t*-test; *p*  < 0.001).

Using paired *t*-test, the serum levels of total protein were significantly decreased (*p* < 0.001) after induction of diabetes in group IV (STZ); however, the AST and ALT levels were significantly increased compared to negative control group I (*p* < 0.001 for both). The total protein levels were significantly improved and increased (*p* < 0.001) to the normal levels with significant reduction of both ALT and AST after treatment of the STZ rats with *N. Sativa* either with seeds (group V) or oil (group VI) (*p* < 0.001 for both). Serum albumin and globulin levels showed no significant changes.

Using paired *t*-test and compared to the negative control rats (group I), diabetic rats (group IV) had significant increase in triglyceride, total cholesterol, and LDL levels; these were significantly reduced in groups V and VI after treatment with *N. Sativa* seeds and oil (*p* < 0.001, *p* < 0.01, and *p* < 0.001, respectively). In contrast, HDL levels were significantly reduced in STZ rats compared to the negative controls, but after treatment with *N. sativa* seeds and oil, it increased and improved, significantly (*p* < 0.05 and *p* < 0.01, respectively).

### 3.2. Histological Results

In this study, microscopic examination of the pancreatic tissue of rats fed with *N. sativa* seeds or oil for 4 weeks revealed significant improvement. The distribution of collagenous fibers around the ducts, vessels, and islets was reduced compared to the STZ group and nearly resembled that of the control group. The shape of pancreatic acini was moderately improved, and most of them showed no vacuolization. Most of the nuclei and zymogen granules looked almost the same as those in the control group. The islets of Langerhans looked similar to those of the control group and had normal densities of *β* cells in the central and peripheral regions of the islet.

**Figure 4:**
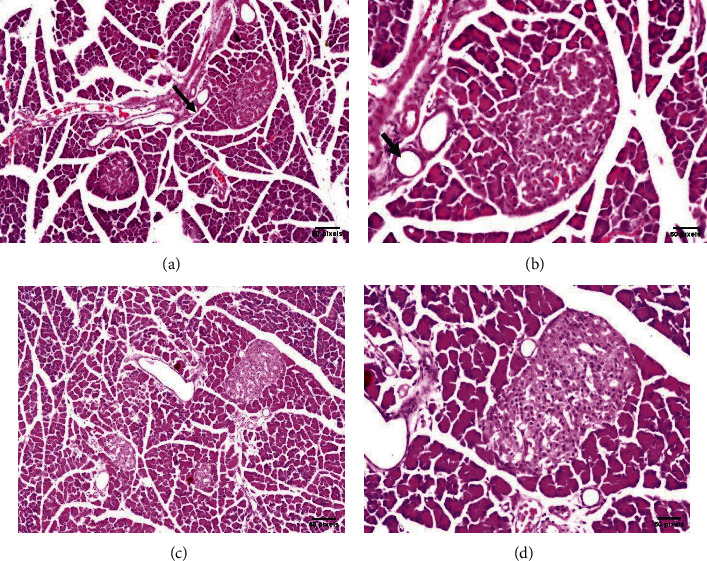
The pancreases of group I rats (negative control) showed a normal histological structure of islets of Langerhans as the endocrine part and the surrounding acini and ductal system of the exocrine part in lobules ((a) H&E ×16; (b) H&E ×40) arrow. In group II rats that administered *N. sativa* seeds, i.e., seeds-treated negative control, the islet of Langerhans showed normal sizes and shapes in most of the lobules ((c) H&E ×16; (d) H&E ×40) arrow.

**Figure 5:**
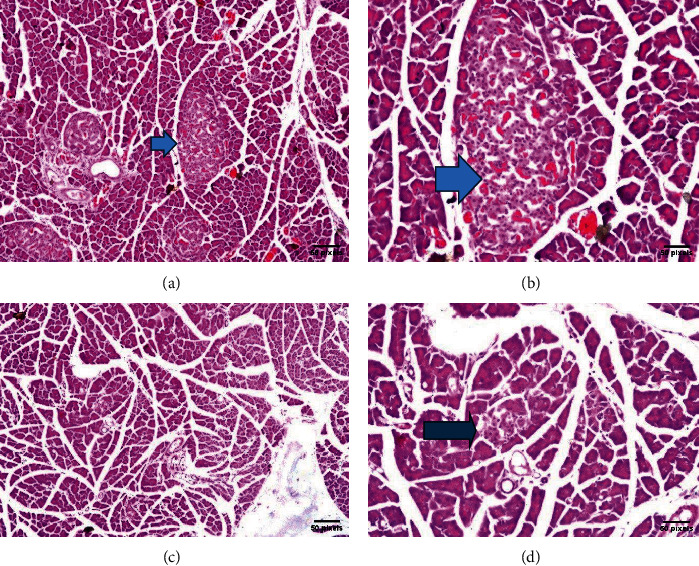
For group III rats (administered *N. sativa* oil, i.e., oil-treated negative control), most lobules of islets of Langerhans showed normal size and shape ((a) H&E ×16; (b) H&E ×40) arrow. For group IV rats (DM-inducted with STZ as a positive control), the islets of Langerhans showed atrophy in most of the lobules ((c) H&E ×16; (d) H&E ×40) arrow.

Groups I, II, and III: The islets of Langerhans appeared as ellipsoidal and spherical structures of varying size and were unequally scattered across the pancreatic acini. The islet is composed of epithelial cells forming trabecular structures that are separated by a dense network of anastomosing capillaries. The shape of the epithelial cells was round/oval containing rounded nuclei in its center, while being surrounded by a delicate cell membrane filled with a fine-grained cytoplasm and tubular formation around the central capillary (Figures 4 and 5).

Group IV (STZ diabetic rat): The islets of Langerhans showed considerable architectural distortion, e.g., being damaged and shrunken in size. The Langerhans *β* cells showed apoptosis with cystic dilatation and luminal eosinophilic deposits in the ductal system (Figure 5).

**Figure 6:**
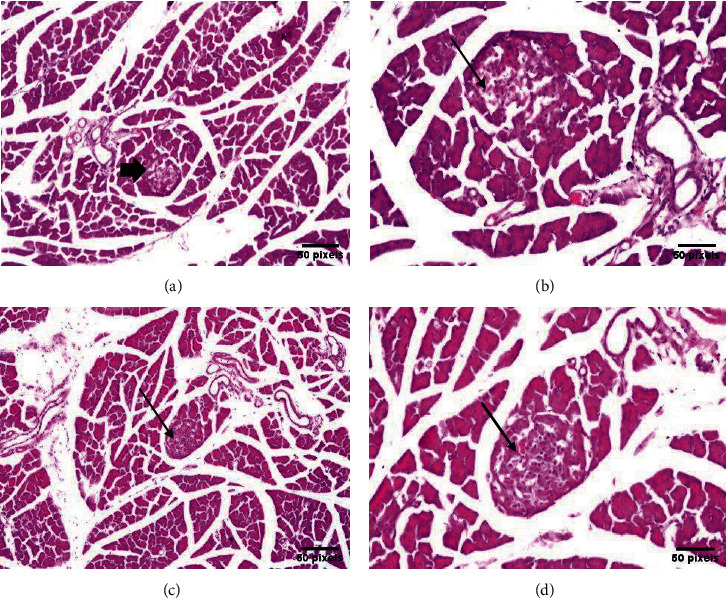
For group V rats (STZ, DM-inducted and treated with *N. sativa* seeds), moderate size and less-than-normal islets of Langerhans cells were observed all over the parenchyma ((a) H&E ×16; (b) H&E ×40) arrow. For group VI rats (STZ, DM-inducted and treated with *N. sativa* oil), moderate and subnormal size and shape were observed in the islets of Langerhans in most lobules ((c) H&E ×16; (d) H&E ×40) arrow.

Groups V and VI: After treatment with *N. sativa* seed or oil, the islets of Langerhans showed considerable recovery in size and shape, with intact cells without cystic dilatation of the ductal system in contrary to group IV (Figure 6).

### 3.3. Immunohistochemical Results

**Figure 7:**
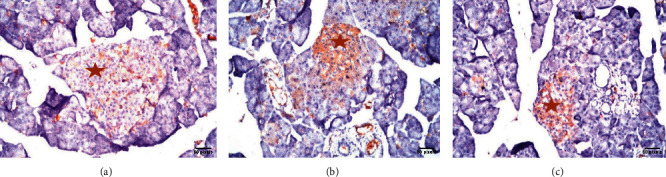
(a) Pancreas section photomicrograph of group I, showing strong positive immunoreaction for insulin in numerous *β* cells, indicated by brown color (anti-insulin antibody reaction; ×40) star. (b) Pancreas section photomicrograph of group II, showing very strong positive immunoreaction for insulin in numerous *β* cells, indicated by brown color (anti-insulin antibody reaction; ×40) star. (c) Pancreas section photomicrograph of group III, showing strong positive immunoreaction for insulin in numerous *β* cells, indicated by brown color (anti-insulin antibody reaction; ×40) star.

**Figure 8:**
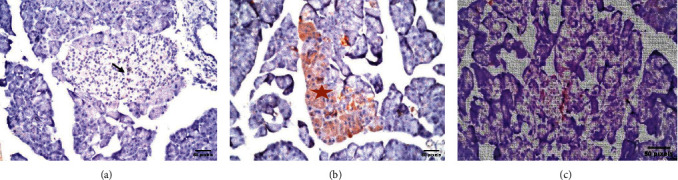
(a) Pancreas section photomicrograph of group IV, showing mild immunoreaction for insulin in few *β* cells, indicated by brown color (anti-insulin antibody reaction; ×40) arrow. (b) Pancreas section photomicrograph of group V, showing moderate immunoreaction for insulin in numerous *β* cells, indicated by brown color (anti-insulin antibody reaction; ×40) star. (c) Pancreas section photomicrograph of group VI, showing moderate immunoreaction for insulin in numerous *β* cells indicated by brown color (anti-insulin antibody reaction; ×40).

The islets of Langerhans of groups I, II, and III showed a very strong reaction in almost all islet cells (Figure 7 and Table 2). In contrast, the islets of Langerhans of group IV showed no reaction in almost all islet cells (Figure 8 and Table 2). Groups V and VI islets of Langerhans showed a moderate and mild reaction in almost all islet cells (Figure 8 and Table 2).

### 3.4. Morphometric Results

The results of morphometric study can be seen in Table 2.

## 4. Discussion

Diabetes mellitus is one of the most common endocrine diseases that affects the human body. It induces various metabolic disorders, including hyperglycemia, hyperlipidemia, hypertension, atherosclerosis, retinopathy, neuropathy, and nephropathy [48, 49, 50]. Controlling DM and preventing its related complications remains a challenge to healthcare professionals and researchers in finding natural products to overcome DM and the long-term adverse effects that gradually develop in diabetic patients. *N. sativa* is one of the most extensively studied natural products; it has a broad range of benefits to human health including its abilities to modify serum glucose and insulin levels, improve cell metabolism and gene regulation, and delay or even prevent early as well as late complications of DM [23].

In the present study, we demonstrated the benefits of *N. sativa* in controlling the blood glucose level, maintaining the integrity of pancreatic beta cells, and improving the lipid profiles, and liver and renal functions in STZ-induced diabetic rat models. However, our study confirms the findings of previous studies that investigated *N. sativa* therapeutic characteristics, but we add that *N. sativa* seeds and oil have nearly the same therapeutic and protective effects on reducing the blood glucose levels, improving the liver and renal functions as well as the lipid profile of the experimented animal models, consistently and time-dependently.

Our findings demonstrated that feeding STZ-diabetic rats with *N. sativa* seeds or oil for 28 days consistently and time-dependently resulted in significant reductions in blood glucose levels. The hypoglycemic effect observed in oil-treated STZ-diabetic rats was comparable to that in seed-treated ones (Table 1). Similar results have been found in several studies using *N. sativa* seeds and oil as antihyperglycemic agents to treat DM [31, 51, 52, 53, 54]. However, the mechanism of the antihyperglycemic effect of *N. sativa* oil and methanolic extract resulted from the inhibition of liver gluconeogenesis pathway enzymes rather than inhibition of intestinal glucose absorption or stimulation of insulin secretion [51]. In the line with our results, a study by Meral et al. [55] involving New Zealand diabetic male rabbit's treated with *N. sativa* for 2 months showed a significant decrease in blood glucose levels. In an Arab clinic, a clinical trial was performed with 94 diabetic patients, who were given oral capsules containing 1, 2, and 3 g doses of powdered *N. sativa* to be taken daily for 12 weeks; only the 2 g/day dose of powdered *N. sativa* resulted in a significant decrease in blood glucose levels [56]. From our results and these previous studies, we can conclude that *N. sativa* preparations can be used effectively for DM management.

Regarding the effect of *N. sativa* seed and oil extracts on the lipid profile, our results showed that treatment of STZ-diabetic rats with *N. sativa* seeds and oil could significantly reduce the high triglyceride, total cholesterol, and LDL serum levels and significantly elevate the HDL levels (Figure 3). However, there were no significant differences between the results of *N. sativa* seeds and those of *N. sativa* oils extracts. These results are consistent with previous studies [57, 58] which mentioned that the oil of *N. sativa* has an antilipidemic effect. In other studies, after treatment with *N. sativa* seed powder or oil, experimental animals with hypercholesterolemia, high levels of triglycerides, LDL, and low levels of HDL showed marked improvement of these lipid profile parameters and showed antiatherogenic cardio-protective properties [59, 60, 61]. The mechanism of *N. sativa* to lower the lipid profile (total cholesterol, triglycerides, LDL-C, and VLDL-C) is thought to be due to its content of monounsaturated fats and phenols. *N. sativa* oil is rich in conjugated linoleic acid, thioquinone, and nigellone dithymoquinone, which have protective antioxidant effects [62].

Clinical trials of *N. sativa* use with human subjects with hyperlipidemia could significantly improve their lipid profiles [63, 64, 65]. For instance, Rao et al. [66] recruited 40 subjects with overweight and DM who consumed two chapatti supplemented with *N. sativa* and Trigonella foenum-graecum for 3 months, this significantly lowered their lipid profile parameters compared to pretreatment figures. In addition, Kaatabi et al. [67] proved that usage of only 1 g/day, *N. sativa* seeds powders produced a significant increase in HDL-C, although usage of 2 g/day *N. sativa* seeds powders produced a significant decline in total cholesterol (TC) TG, and LDL-C, as well as a significant elevation in HDL-C/LDL-C. Therefore, a mere dose of 2 g/day of *N. sativa* for 12 weeks might improve dyslipidemia associated with type 2 DM. Consequently, these studies suggested that supplements with *N. sativa* preparations can improve complications associated with the lipid and lipoprotein profiles of patients with DM, depending on the dose and the duration of the intervention [67, 68]. In the present study, *N. sativa* seed and oil extracts also demonstrated marked improvement of both liver and renal functions in STZ-diabetic rats. Similarly, a study by Coban et al. [69] showed a decrease in AST, ALT, and activities of the *N. sativa*-treated rats compared to controls (*p* < 0.05 for both ALT and AST).

In a similar study, treatment of diabetic rats with *N. sativa* oil for 8 weeks significantly decreased the liver enzymes (ALT, AST, and ALP) as well as the kidney parameters (urea, creatinine, and uric acid) compared with the positive control group, returning them to the normal levels [61]. These results are congruent with ours except for the creatinine levels which were decreased but did not reach a level of significance. This can be explained by the period of the treatment with *N. sativa* which lasted for 8 weeks in the prior study compared to only 4 weeks in ours. A separate study used *N. sativa* extracts to examine its protective value on the liver and kidney against the damaging effects of lipopolysaccharide (LPS)-treated rats; the results showed that the negative effects of LPS on AST, ALT, serum proteins, urea, and creatinine were reversed to normal levels after treatment with *N. sativa* extracts [70]. Our findings support the previous research, confirming that *N. sativa* extracts are a natural product that prevents the damaging effects of diabetes on the liver and kidney functions of experimental animals.

In the present study, the histology of the STZ-induced rats showed necrotic and degenerative changes in the central part of the islets of Langerhans. These findings are similar to those of earlier studies [71, 72]. Massive increase in the concentration of cytosolic calcium decreased insulin secretion because of the rapid damage of the *β* cells of Langerhans. Our histopathological results of the STZ-diabetic rats showed a noticeable reduction in the size of the pancreatic *β* cells' cellular components with varying degrees of degenerative changes and apoptosis. These results are consistent with that of a previous study that reported [73] a significant decrease in the pancreatic *β* cells density in diabetic rats.

Numerous studies have confirmed the ability of *N. sativa* to maintain the integrity of pancreatic *β* cells, induce lipid peroxidation, and increase the activity of the antioxidant defense system [52, 53, 55, 74]. Kanter et al. [75] studied the effect of *N. sativa* essential oil on the histopathology of pancreatic *β* cells in diabetic rats and found that treatment with *N. sativa* decreased elevated serum glucose, increased insulin concentration, and partially regenerated pancreatic *β* cells in the animals. Subsequently, they showed that the positive effect of *N. sativa* on the number of *β* cells was accompanied by a reduction in lipid peroxidation and an increase in antioxidant enzyme activity [76, 77].

In contrast, a study conducted by Ikram and Hussain [78] showed that *N. sativa* extracts did not regenerate the *β* cells of pancreatic islets in alloxan-induced diabetic male rabbits. However, these inconsistent results can be attributed to the short duration of their experiment as they fed the animals for only 2 weeks which could be the reason for the insignificant response to their intervention [78].

In conclusion, *N. sativa* whole seeds or oil extracts have significant control on the serum glucose levels of diabetic rats and can improve the negative impacts of DM on lipid profiles, liver, and renal functions. Moreover, *N. sativa* extracts were effective in protecting the islets of Langerhans through significant high antidiabetic and rejuvenating effects on tissues.

## Figures and Tables

**Table 1:** Effects of *N. sativa* seeds and oil on blood glucose levels at days 0, 7, 14, and 28 in all studied groups (STZ-induced diabetic rats and seeds and oil-treated rats).

Groups	Glucose (mg/dl)			
	Day 0 Mean ± SE	Day 7 Mean ± SE	Day 14 Mean ± SE	Day 28 Mean ± SE
Group I (negative control)	99.1 ± 0.3	99.4 ± 0.3	99.6 ± 0.4	99.7 ± 0.4
Group II (normal + seeds)	100.7 ± 0.5	100.9 ± 0.5	100.9 ± 0.5	101.0 ± 0.5
Group III (normal + oil)	99.7 ± 0.4	99.7 ± 0.4	99.7 ± 0.4	99.8 ± 0.4
Group IV (STZ)	99.7 ± 0.2	414.1 ± 1.6	414.5 ± 1.7	414.8 ± 1.3
Group V (STZ + seeds)	100.0 ± 0.3	408.3 ± 1.7	234.5 ± 0.6 ^*∗*^	156.3 ± 0.8 ^*∗*^
Group VI (STZ + oil)	98.3 ± 0.3	412.2 ± 1.4	233.3 ± 1.1 ^*∗*^	157.1 ± 0.4 ^*∗*^

Unpaired *t*-test was used to compare the blood glucose levels within each group on weekly basis; however, paired *t*-test was used to compare means of the blood glucose levels between groups.  ^*∗*^*p* < 0.01 for Groups V and VI compared to Group IV, on day 14.  ^*∗*^*p* < 0.001 for Groups V and VI compared to Group IV, on day 28.

**Table 2:** The quantitative measurements of mean gray of insulin content.

Group	Group I Negative control	Group II Seed	Group III Oil	Group IV STZ	Group V STZ + seed	Group VI STZ + oil
Anti-insulin antibody	++++	++++	++++	−	+++	++

++++ indicates very strong reaction; +++ indicates strong reaction; ++ indicates moderate reaction; + indicates mild reaction; and − indicates no reaction.

## Data Availability

Data and materials of this study are openly available.

## Additional Points

*Study Limitations*. The findings of this study are based mainly on the experimental results of using *N. sativa* on diabetic animal models, and it has not been verified on human volunteers, yet.
